# Are prefabricated foot orthoses still effective in reducing plantar pressures after 12 months of wear?

**DOI:** 10.1186/1757-1146-4-S1-O13

**Published:** 2011-05-20

**Authors:** Dean G Cronkwright, Martin J Spink, Karl B Landorf, Hylton B Menz

**Affiliations:** 1 Department of Podiatry, Faculty of Health Sciences, La Trobe University, Victoria 3086, Australia; 2 Musculoskeletal Research Centre, Faculty of Health Sciences, La Trobe University, Bundoora, Victoria 3086, Australia

## Background

Foot problems are highly prevalent in older people, and prefabricated (‘off-the-shelf’) foot orthoses are frequently prescribed in this age-group. However, such devices are susceptible to material compression and deformation, which may reduce their effectiveness over time. Therefore, the aim of this study was to investigate the difference in plantar pressures between prefabricated orthoses worn for at least 12 months to new prefabricated orthoses in older people.

## Methods

Thirty-one adults aged over 65 years who were taking part in a larger falls prevention trial were recruited (21F, 10M, mean age 75.4+/- 5.2 years). Plantar pressure data was recorded using the PEDAR® in-shoe system while participants walked along an eight metre walkway wearing shoes only, new prefabricated orthoses and orthoses they had been wearing for at least 12 months. The orthoses used were Formthotics® dual density red. Pressure data (peak pressure, maximum force and contact area) were collected for the forefoot, midfoot, and rearfoot.

## Results

The new and old orthoses functioned similarly, with both devices significantly reducing peak pressures at the heel (new=23%, old=18%) compared to the shoe only condition. The reduction in peak pressures was achieved by corresponding increases in maximum force at the midfoot (new=42%, old=44%), and contact area at the midfoot (new=51% and old=53%) and forefoot (new=11%, old=13%) compared to the shoe only condition. There were no significant differences between the old and new orthoses, with the exception of small but statistically significant increases in peak pressure at the heel (6%) and maximum force at the heel (5%) and forefoot (2%) in the old orthoses compared to the new orthoses.

## Conclusions

Prefabricated foot orthoses worn by older people for at least 12 months are similarly effective at reducing plantar pressures compared to new orthoses, indicating there is only limited deformation over a 12 month period. Therefore, in this age-group, there may not be a need to replace the orthoses as frequently as previously thought.

